# Diagnostic Value of *DAPK* Methylation for Nasopharyngeal Carcinoma: Meta-Analysis

**DOI:** 10.3390/diagnostics13182926

**Published:** 2023-09-12

**Authors:** Thuan Duc Lao, Phuong Kim Truong, Thuy Ai Huyen Le

**Affiliations:** Department of Pharmaceutical and Medical Biotechnology, Faculty of Biotechnology, Ho Chi Minh City Open University, Ho Chi Minh City 700000, Vietnam; phuong.tk@ou.edu.vn (P.K.T.); thuy.lha@ou.edu.vn (T.A.H.L.)

**Keywords:** *DAPK*, nasopharyngeal carcinoma, diagnosis, meta-analysis, methylation

## Abstract

Background: Methylation of *DAPK* has been reported to play a key role in the initiation and progression of nasopharyngeal cancer. However, there are differences between the studies on it. This meta-analysis was performed to evaluate the diagnostic value of *DAPK* promoter methylation for NPC. Method: The study method involves the systematic research of eligible studies based on criteria. The frequency, odds ratios (OR), sensitivity as well as specificity with the corresponding 95% confidence intervals (CIs) were used to assess the effect sizes. Results: A total of 13 studies, including 1048 NPC samples and 446 non-cancerous samples, were used for the meta-analysis. The overall frequencies of *DAPK* methylation were 56.94% and 9.28% in NPC samples and non-cancerous samples, respectively. The association between DAPK methylation and risk of NPC was also confirmed by calculating the OR value which was 13.13 (95%CI = 54.24–40.72) based on a random-effect model (Q = 64.74; *p* < 0.0001; I^2^ = 81.47% with 95%CI for I^2^ = 69.39–88.78). Additionally, the study results suggest that testing for *DAPK* methylation in tissue samples or brushing may provide a promising method for diagnosing NPC. Conclusion: This is the first meta-analysis that provided scientific evidence that methylation of the *DAPK* gene could serve as a potential biomarker for diagnosis, prognosis, and early screening of NPC patients.

## 1. Introduction

Nasopharyngeal carcinoma (NPC), also known as nasopharyngeal cancer, is a type of human cancer which originates from mucosal epithelium of the nasopharynx, the upper part of the throat behind the nose. NPC has been reported to be a relatively rare type of malignant tumor, but it is remarkably distributed geographically towards Southeast Asia, including southern China, Hong Kong, and Southeast Asian countries [1]. According to GLOBOCAN (Global Cancer Observatory) (2020), NPC is a significant global health concern, with variations in its incidence and mortality rates across different regions. The highest incidence rates of NPC are observed in certain populations, particularly in Southeast Asia, including southern China, Hong Kong, and Southeast Asian countries like Malaysia, Indonesia, and Singapore. In detail, 133,354 new nasopharyngeal cases were recorded in the world, of which 80,008 died [2].

Due to the indistinct nature of NPC symptoms, individuals may experience many persistent symptoms such as a sore throat, nasal congestion, nosebleeds, difficulty in breathing or speaking, hearing loss, and facial pain or numbness. Furthermore, due to its deep location within the head and neck, nasopharyngeal carcinoma has the potential to spread to neighboring lymph nodes and distant organs like the lungs and bones. As a result, NPC is frequently detected when it is already progressed [3]. Diagnosing NPC in its advanced stage reduces the success of its treatment as well as the survival of NPC patients [3,4,5]. The early diagnosis and screening of patients with NPC can extend or increase their overall survival. As previously mentioned, the obstacles to the early diagnosis and screening are non-specific symptoms that occur during the early stage of NPC and the deeply seated location of the nasopharynx [4,6]. Therefore, effective biomarkers are truly needed. There is unanimous consensus on the use of biomarkers for early diagnosis and screening of NPC. There is complete agreement among experts and researchers in the medical community regarding the effectiveness and importance of using biomarkers, including the infection of Epstein–Barr virus (EBV), genetic modification, etc., as diagnostic tools for early detection and screening of NPC [7,8]. Currently, many studies have demonstrated that the phenomenon of tumor suppressor gene (TSG) promoters causes transcriptional silencing. This inactivates and inhibits the functions of those genes, resulting in tumorigenesis. In addition, methylation occurs early in molecular alterations and malignant transformations in human epithelial cells [9]. Thus, in the context of diagnosing and screening for NPC, the patterns of TSG promoter methylation for detecting methylated tumor suppressor genes have been postulated as the best-studied and most extensively epigenetic biomarker for diagnosing and screening NPC [10,11,12]. By examining the methylation status of specific TSG promoters, it is possible to identify methylated tumor suppressor genes, which can serve as indicators for the presence or progression of NPC. This approach holds promise for early detection and monitoring of the disease.

Death-associated protein kinase (*DAPK*), located on chromosome 9q34.1, encodes a stress-regulated Ser/Thr protein kinase which plays well-documented roles in the regulation of cellular signaling pathways with diverse outcomes, including apoptosis, autophagy, and immune responses [13,14]. Epigenetic modifications, especially promoter hypermethylation, are the primary factors responsible for the inactivation of *DAPK* in NPC [15,16,17,18]. DAPK has been reported as the mediator of cell death of INF-γ-induced apoptosis. Additionally, DAPK is essential for activation of various cell death mechanisms, caspase dependent or not, and the p19ARF/p53 signaling pathway [19,20,21]. Thus, the decreased expression of DAPK was associated with the methylation of its promoter, leading to tumorigenesis. Even though the methylation of *DAPK* has been widely studied, there is still a patchy and inconsistent picture of *DAPK* because the studies differ greatly. These differences were caused by the different sensitivities and intra/inter-assay coefficients of variation of methods, the source of samples as well as the populations. For example, Fendri et al. (2009) reported that the frequency of DAPK in NPC was 88.24% [15]. Contrary to Frendri et al. (2009), Nawaz et al. (2015) observed a low frequency of 25.0% in their study [16]. Therefore, in the current study, a comprehensive meta-analysis was conducted to evaluate and determine the diagnostic value of *DAPK* methylation in NPC. This approach allowed for the systematic review and synthesis of existing evidence from multiple independent studies, aiming to provide a more reliable assessment of the diagnostic potential of *DAPK* methylation in NPC.

## 2. Materials and Methods

### 2.1. Search Strategies and Inclusion/Exclusion Criteria

Detailed and comprehensive research was conducted in the following databases: PubMed, Web of Science, and Scopus, and all related published studies, based on the guidelines of Preferred Reporting Items for Systematics Reviews and Meta-Analyses. Our study team used the following keywords and MeSH terms in conjunction with a highly sensitive search strategy: [“epigenetics” or “epigenomics”] and [“methylation” or “DNA methylation” or “hypermethylation” or “promoter methylation” or “promoter hypermethylation” or “DNA hypermethylation”] and [“nasopharyngeal carcinoma” or “nasopharyngeal cancer” or “nasopharynx cancer” or “NPC”] and [“*DAPK*” or “*DAPK1*” or “*Death-associated protein kinase*”]. We also conducted a manual search to find other potential articles based on references identified in the individual articles.

Studies were considered eligible only when they met all of the following inclusion criteria: (i) The articles were limited to studies written in English; (ii) they must have case–control study design; (iii) the design must be focused on the relationship between *DAPK* methylation and nasopharyngeal tumorigenesis; (iv) the study must provide sufficient information about the frequencies of *DAPK* promoter methylation; (v) studies should provide information on the assessment or measurement of DAPK methylation. This can include techniques such as bisulfite sequencing, methylation-specific PCR, or pyrosequencing. Those studies that could not meet the inclusion criteria were excluded. The exclusion criteria were as follows: (i) Articles written in other languages; (ii) articles containing abstracts, case reports, letters to the editor, or unpublished articles were eliminated; (iii) irrelevant studies: Studies related to other tumors and not specific to NPC; (iv) studies that lacked vital information for analysis; (v) duplicate or overlapping studies: If multiple studies present the same dataset or share significant overlap in terms of study participants, data, or methodology, only the most comprehensive or recent study may be included to avoid duplication.

### 2.2. Data Extraction

The relevant data were systematically extracted and individually retrieved by two authors using a standardized form to ensure consistency and accuracy. The form used for the extraction documented the most relevant items, including the name of the first author, publication year, geographical location, sample size, the source of samples, methylation frequencies, and detection method of methylation.

### 2.3. Statistical Analysis

The MedCalc^®^ software version 22.009, owned by MedCalc Software Ltd. (Ostend, Belgium), was applied to statistically analyze the extracted data. Odds ratio (OR) with 95% confidence interval (95%CI) was calculated and computed in forest plots to evaluate the strength of *DAPK* methylation for the risk of NPC.

The Cochran Q test and I^2^ statistics were applied to assess the heterogeneity among the studies [15]. A significance level of *p* = 0.05 was used as the cut-off point for both the Q test and I^2^ to determine the presence of heterogeneity between the studies. The scale of the I^2^ value was employed to classify the degree of heterogeneity: I^2^ < 25% indicated no heterogeneity, 25% ≤ I^2^ ≤ 50% indicated moderate heterogeneity, and I2 > 50% indicated strong heterogeneity. The random-effect model was applied if heterogeneity existed among the studies (*p* < 0.05 for Q test, I^2^ > 50%). Where there was no heterogeneity between studies, a fixed-effects model was applied to compute the pooled ORs (I^2^ < 50%). In order to determine if there was publication bias, the symmetry of the funnel plots in which ORs were plotted against their corresponding standard errors was assessed by the Begg’s funnel plot and Egger’s test. *p <* 0.05 indicates statistical significance [22,23,24,25,26]. Additionally, sensitivity analysis was performed by sequentially omitting individual studies to evaluate the stability of their results.

## 3. Results

### 3.1. Identification of Extracted Studies

**Figure 1:**
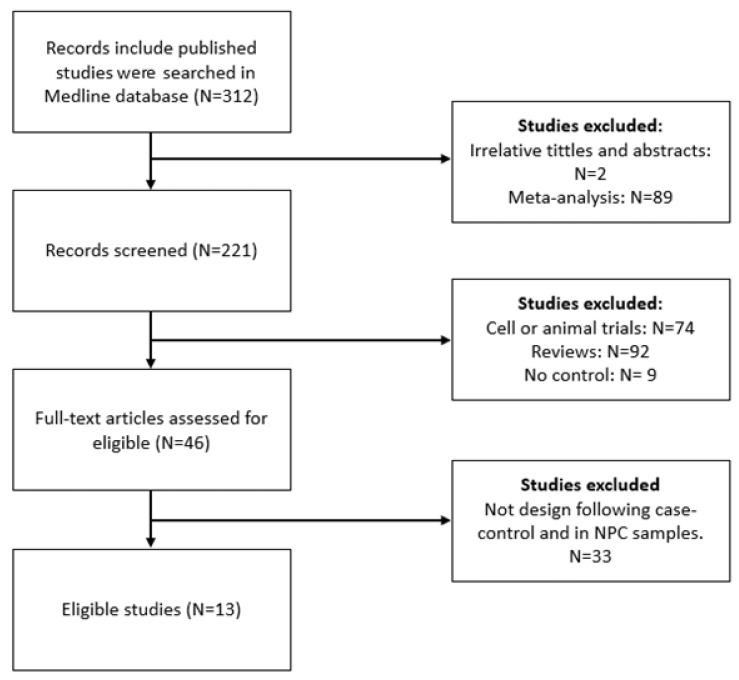
Flow chart of study selection in the meta-analysis.

The selection and screening process of the relevant studies are illustrated in the PRISMA flow chart (Figure 1). The characteristics of the relevant studies included are summarized in Table 1.

Following a rigorous screening process based on the previously mentioned inclusion criteria, a total of 13 studies were deemed eligible for inclusion in the current meta-analysis. These 13 relevant studies collectively encompassed a sample size of 1492 samples, of which 1048 derived from NPC patients and 446 samples from non-cancerous samples, encompassing publication years from 2002–2015. Regarding the ethnicity of the patients included in the 13 studies, the majority of the studies (10 out of 13, accounting for 76.92%) focused on individuals from Asian countries. The remaining three studies (23.08%) included patients from African countries. This distribution reflects the regional focus of the research and the representation of different ethnic backgrounds within the selected studies. Regarding the evaluation of *DAPK* methylation, out of the 13 eligible studies, 11 studies (84.62%) specifically examined the methylation status of the *DAPK* gene in samples obtained from NPC biopsies. This indicates a predominant emphasis on analyzing tissue samples for *DAPK* methylation. Furthermore, four studies (accounting for 30.77%) evaluated the correlation between methylation of *DAPK* and NPC in plasma (case) versus plasma (control) samples. This approach offers a non-invasive method for assessing *DAPK* methylation status and its potential diagnostic or prognostic value in NPC. In terms of the test method employed for evaluating the status of *DAPK* methylation, the majority of the studies (10 out of 13, accounting for 76.92%) utilized the methylation-specific PCR (MSP) method.

### 3.2. Association between Methylation of DAPK Promoter and NPC in NPC versus Non-Cancerous Samples

**Figure 2:**
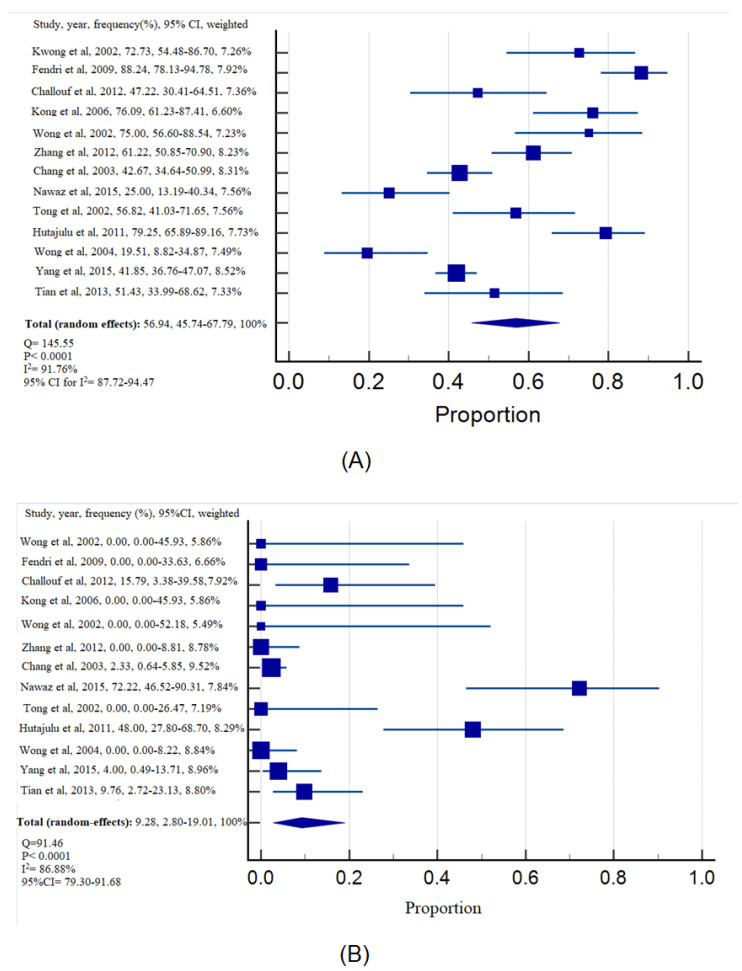
Forest plot of *DAPK* gene’s methylation frequency in (**A**) NPC samples, (**B**) non-cancerous samples.

**Figure 3:**
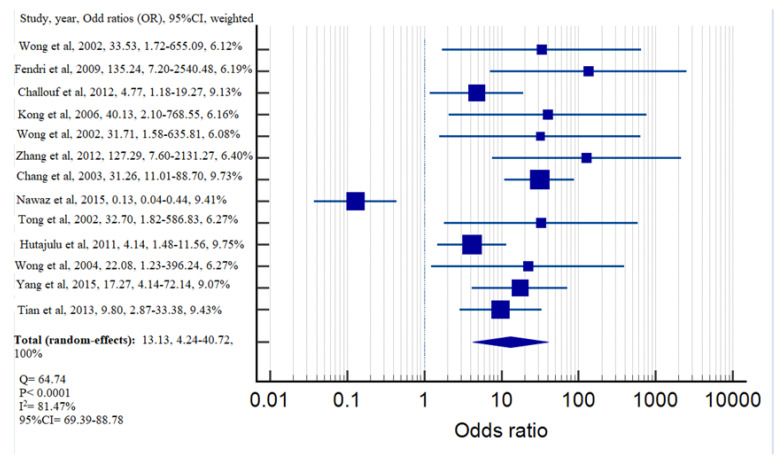
Forest plot of *DAPK* promoter methylation associated with NPC by evaluating odds ratio risk using random-effect model.

Figure 2 shows the frequency of *DAPK* gene methylation among samples obtained from individuals with NPC and non-cancerous samples. Regarding the heterogeneity between the studies, there was significant heterogeneity across studies in the case group (Q = 144.55, *p* < 0.0001, I^2^ = 91.76%, 95%CI for I^2^ = 87.72–94.47) and control group (Q = 91.46, *p* < 0.0001, I^2^ = 86.88%, 95%CI for I^2^ = 79.30–91.68), highlighting substantial variations between the studies. Consequently, the random-effect model was applied to calculate the frequency of *DAPK* gene methylation in NPC and non-cancerous samples. The weighted frequencies of *DAPK* gene methylation in NPC and non-cancerous samples were 56.94% and 9.28%, respectively. The methylation of the *DAPK* gene was associated with an increased NPC risk with pooled OR of 13.13, based on the random-effect model (Q = 64.74, *p* < 0.0001, I^2^ = 81.47%, 95%CI for I^2^ = 69.39–88.78) (Figure 3).

In order to further explore the potential impact of various factors on the relationship between *DAPK* gene methylation and the risk of nasopharyngeal carcinoma (NPC), subgroup analyses were conducted based on ethnicity, source of sample, and test method, and their respective outcomes are presented in Table 2. The subgroup analysis based on ethnicity revealed that methylation of the *DAPK* promoter significantly correlated with an increased risk of NPC in African countries (OR = 3.57, 95%CI = 0.10–126.16, *p* < 0.0001). This indicates that *DAPK* gene methylation is particularly relevant to the development of NPC in individuals of African descent. Additionally, when the analysis was stratified based on the source of NPC samples, it was observed that there were strong associations between *DAPK* promoter methylation and NPC among individuals whose samples were obtained through NPC biopsy tissue (OR = 15.32, 95%CI = 1.86–125.93, *p* < 0.0001), indicating that *DAPK* gene methylation is highly indicative of NPC risk when assessed using biopsy tissue samples. Furthermore, the subgroup analysis based on the test method employed to detect *DAPK* gene methylation demonstrated a strong relationship between methylation of the DAPK promoter and NPC when the methylation-specific polymerase chain reaction (MSP) test method was used (OR = 10.35, 95%CI = 2.71–39.53, *p* < 0.0001).

### 3.3. Diagnostic Value of DAPK Methylation

**Figure 4:**
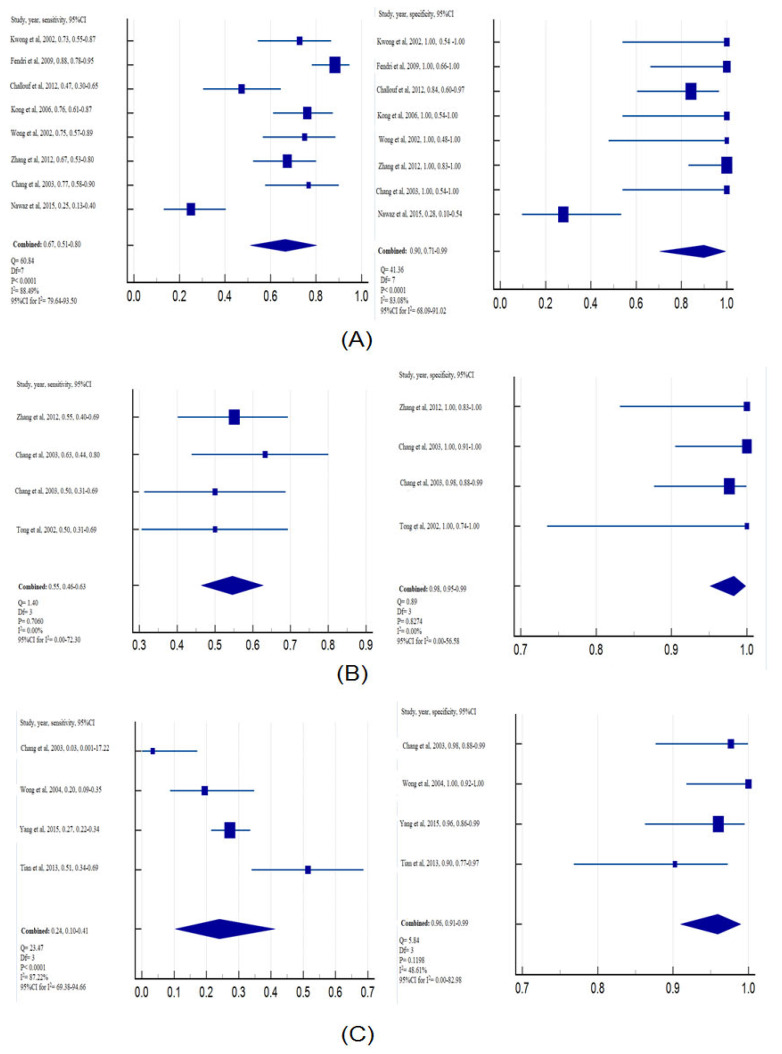
Forest plot of sensitivity and specificity of *DAPK* promoter methylation associated with NPC: (**A**) Tissue samples; (**B**) brushing samples; (**C**) plasma samples.

In order to assess the diagnostic capability of *DAPK* promoter methylation, a comparative analysis was conducted on different sample types, including biopsy, brushing, and blood, obtained from both individuals with NPC and control samples. For the source of tissue, the pooled sensitivity and specificity of *DAPK* promoter methylation in the tissue samples were calculated to be 0.67 (95%CI = 0.51–0.80) and 0.90 (95%CI = 0.71–0.99), respectively (Figure 4A). Moving to brushing samples, the pooled sensitivity and specificity of *DAPK* promoter methylation were 0.55 (95%CI = 0.46–0.63) and 0.98 (95%CI = 0.95–0.99), respectively (Figure 4B). Additionally, the overall sensitivity and specificity of *DAPK* promoter methylation in plasma samples were 0.24 (95%CI = 0.10–0.41) and 0.96 (95%CI = 0.91–0.99), respectively (Figure 4C).

## 4. Discussion

The methylation of tumor suppressor genes represents a crucial molecular mechanism in the era of epigenetic regulation, exerting a significant influence on the process of human tumorigenesis [38,39,40]. The aberrant methylation of the *DAPK* promoter has emerged as a prominent factor implicated in the initiation and progression of NPC. By promoting the silencing of the *DAPK* gene, this abnormal methylation event disrupts its normal functioning and triggers a cascade of biological events that contribute to the development and advancement of NPC [15,16]. Increasing evidence has suggested that aberrant methylation of the *DAPK* promoter, which leads to its silencing, is responsible for the initiation and progression of NPC through the regulation of many signaling pathways, including apoptosis, autophagy, and immune responses [15,16]. The silencing of *DAPK* due to aberrant methylation impedes the apoptotic signaling cascade, leading to uncontrolled cell proliferation and evasion of cell death, which are hallmarks of cancer development. However, there persist inconsistencies and heterogeneities among previous reports regarding this methylation. This variation can be observed in the wide range of reported methylation frequencies of the *DAPK* promoter in NPC, spanning from 25.0% to 88.24% in different studies [15,16]. These disparities raise the need for a comprehensive and systematic investigation of the association between *DAPK* promoter methylation and NPC to gain a more precise understanding of its role in the development and progression of the disease.

To address these discrepancies, the present study employed a systematic review and meta-analysis approach. This methodology provides a robust and rigorous framework for synthesizing data from multiple independent studies, enabling a comprehensive evaluation of the association between *DAPK* promoter methylation and NPC. By systematically collecting relevant studies and subjecting them to rigorous analysis, it can generate more accurate and representative estimates of the diagnostic performance of a particular biomarker, herein, *DAPK* methylation, in NPC. Generally, the current study aimed to elucidate the overall relationship between *DAPK* promoter methylation and NPC, while also exploring potential sources of heterogeneity among the existing literature.

Based on the analysis of *DAPK* promoter methylation in 1048 NPC samples and 446 non-cancerous samples, it is indicated that the presence of the *DAPK* promoter was significantly higher in NPC samples than in non-tumorous samples (NPC: 56.94% vs. non-tumorous samples: 9.28%) (Figure 2). These results, as illustrated in Figure 2, clearly highlight the substantial association between *DAPK* promoter methylation and the presence of NPC. Moreover, the individuals whose *DAPK* gene was methylated were significantly associated with NPC from the calculation of pooled OR using the random-effect model (OR = 13.13, 95%CI = 4.24–40.72) (Figure 3). The resulting OR indicated that there was a 13.13-fold increase in the odds of a positive outcome in *DAPK* methylation, and this increase was statistically significant at the 5% level. These compelling results strongly support the pivotal role of DAPK promoter methylation in the tumorigenesis of NPC. The significant difference in methylation frequency between NPC and non-tumorous samples, coupled with the markedly increased odds of positive outcomes associated with *DAPK* methylation in NPC, underscores the importance of this epigenetic alteration in the development and progression of the disease. The findings also provide compelling evidence that *DAPK* promoter methylation serves as a crucial molecular marker in NPC, highlighting its potential as a diagnostic and prognostic indicator for the disease. Furthermore, these results emphasize the significance of *DAPK* and its methylation status as potential targets for therapeutic interventions aimed at managing and combating NPC.

The subgroup analysis based on ethnicity which compared the methylation of *DAPK* in NPC samples and non-cancerous samples revealed that African countries had increased risk of NPC (OR = 3.57, 95%CI = 0.10–126.16, *p* < 0.0001). This observation suggests that the African population may be more susceptible to *DAPK* promoter methylation. However, the result of the subgroup analysis based on population should be deeply and carefully considered as only small sample sizes were included in the current study. The subgroup analysis based on the source of cancer samples revealed a significant correlation between *DAPK* promoter methylation and NPC in NPC tissues. It indicates that the type of biopsy was more suitable to evaluate the methylation of the *DAPK* gene. Moreover, the high frequency of *DAPK* methylation in non-invasive samples, including brushing, and plasma samples shows a great potential to be applied as an invasive biomarker for early detection of NPC. Regarding the value of sensitivity and specificity, the value of specificity was not significantly different; the sensitivity of the tissue and brushing samples (tissue: 0.67; brushing: 0.67) was higher than that of the plasma samples (a weak sensitivity: 0.24). Therefore, these results suggest that testing for *DAPK* methylation in tissue samples or brushing may provide a promising method for diagnosing NPC. Hence, in order to enhance sensitivity, it is essential to integrate it with other biomarkers or explore additional patterns of tumor gene suppressor genes. Consequently, future efforts should prioritize the discovery of non-invasive or minimally invasive biomarkers for NPC. Finally, we also found that the MSP method was used in 10 studies (accounting for 76.92%) to evaluate the status of *DAPK* gene methylation. MSP, a useful tool that is easy to design, exhibiting high sensitivity in detecting small amounts of methylated DNA, has been considered as the “gold standard method” for evaluating the status of methylation [41]. The product of MSP analysis is easily to detect through the assay of electrophoresis [42]. Our findings are consistent with the documented *DAPK* gene methylation, which is a frequent occurrence and plays a role in the early epigenetic events of NPC tumorigenesis. Therefore, this epigenetic event has the potential to serve as a valuable biomarker for diagnosing and early screening of NPC. Overall, the characteristics of the included studies provide valuable insights into the sample size, patient ethnicity, sample types, and test methods employed for evaluating DAPK methylation in the context of NPC. These details contribute to the comprehensive understanding of the meta-analysis and form the basis for the subsequent analysis and interpretation of the findings.

In summary, the characteristics of the included studies in this meta-analysis provide valuable insights into sample size, patient ethnicity, sample types, and test methods employed for evaluating *DAPK* methylation in the context of NPC. These details enhance the comprehensiveness of the meta-analysis and form the foundation for subsequent analysis and interpretation of the findings. Collectively, the results support the potential of *DAPK* promoter methylation as a promising epigenetic biomarker for the diagnosis, early screening, and management of NPC. However, further research with larger and more diverse populations is needed to validate these findings and explore additional non-invasive biomarkers for NPC.

This current meta-analysis offered several advantages that enhance the validity and significance of the findings. First, the studies included were case–control. Case–control studies are particularly useful in investigating the association between exposure (*DAPK* promoter methylation) and outcome (NPC), as they provide a suitable framework for evaluating the diagnostic value of a biomarker. Secondly, the current study provided in-depth investigation of the evaluation of the association between *DAPK* promoter methylation and the risk of NPC. Finally, the reliable conclusion was generated via the systematically reviewing, analyzing as well as synthesizing of data from the available evidence. Nevertheless, the present meta-analysis is subject to certain limitations, primarily stemming from the limited number of studies included. One noteworthy concern is the potential bias introduced by the exclusion of non-English language studies’ data. Secondly, most of the studies were performed in Asian countries. Other populations were insufficient, such as Europe, America, etc. Thirdly, in this study, we assessed the relationship between *DAPK* gene methylation and clinicopathological features, age, and TNM stage, along with the differences in DAPK methylation between the case and control groups. Our focus was solely on reporting the methylation status of the *DAPK* gene for this research. However, in future investigations, it would be beneficial to explore methylation patterns of additional candidate genes.

## 5. Conclusions

To sum up, this is the first meta-analysis investigating the association between *DAPK* methylation and NPC. The results of this study provide compelling evidence supporting a significant association between *DAPK* gene methylation and the risk of NPC, as demonstrated by the calculated odds ratios (ORs). The analysis highlights the diagnostic potential of DAPK promoter methylation in tissue and brushing samples. The higher sensitivity observed in these sample types suggests that they may serve as reliable methods for the clinical diagnosis of NPC. These findings pave the way for the development of effective diagnostic approaches that utilize *DAPK* methylation as a molecular marker. In the future, there should be a focus on conducting well-designed clinical studies with larger sample sizes as well as exploring the underlying mechanisms through cell-based investigations. By addressing these research gaps, we can further validate and solidify the role of *DAPK* methylation as an informative biomarker for NPC.

## Figures and Tables

**Table 1:** The characteristics of the eligible studies considered in this report.

Authors	Year	Region	Method	Source of Sample		Case		Control	
				Case	Control	P	N	P	N
Kwong et al. [27]	2002	Asia	MSP	Biopsy	Epithelium	24	33	0	6
Wong et al. [28]	2002	Asia	MSP	Biopsy	Biopsies, epithelium	24	32	0	5
Tong et al. [29]	2002	Asia	MSP	Biopsy	Brushing	11	16	0	12
Tong et al. [29]	2002	Asia	MSP	Brushing	Brushing	14	28	-	-
Chang et al. [30]	2003	Asia	MSP	Biopsy	Biopsies	23	30	0	6
Chang et al. [30]	2003	Asia	MSP	Brushing	Brushing	19	30	0	37
Chang et al. [30]	2003	Asia	MSP	Mouth and throat rising fluid	Mouth and throat rising fluid	15	30	1	43
Chang et al. [30]	2003	Asia	MSP	Plasma	Plasma	1	30	1	43
Chang et al. [30]	2003	Asia	MSP	Buffy coat	Buffy coat	6	30	2	43
Wong et al. [31]	2004	Asia	RT-qPCR	Plasma	Plasma	8	41	0	43
Kong et al. [32]	2006	Asia	MSP	Biopsy	Biopsies	35	46	0	6
Fendri et al. [15]	2009	Africa	MSP	Biopsy	Epithelium	60	68	0	9
Hutajulu et al. [33]	2011	Asia	MSP	Biopsy	Brushing	42	53	12	25
Challouf et al. [34]	2012	Africa	MSP	Biopsy	Biopsies	17	36	3	19
Zhang et al. [35]	2012	Asia	MMSP	Biopsy	Biopsies	33	49	0	20
Zhang et al. [35]	2012	Asia	MMSP	Brushing	Brushing	27	49	0	20
Tian et al. [36]	2013	Asia	MSP	Plasma	Plasma	18	35	4	41
Nawaz et al. [16]	2015	Africa	MSP	Biopsy	Biopsies	11	44	13	18
Yang et al. [37]	2015	Asia	MS-HRM	Plasma	Plasma	60	220	2	50
Yang et al. [37]	2015	Asia	MS-HRM	Biopsy	Plasma	28	52	-	-
Yang et al. [37]	2015	Asia	MS-HRM	Brushing	Plasma	66	96	-	-

P: Positive, N: Sample size, MSP: Methylation-specific PCR, MMSP: Multiplex methylation-specific PCR, RT-qPCR: Real-time quantitative PCR, MS-HRM: Methylation-sensitive high-resolution melting.

**Table 2:** The subgroup analysis for the association between *DAPK* promoter methylation and NPC by ethnicity, source of sample, and test method.

Group	Case		Control		Model, OR, 95%CI	Heterogeneity	
	P	N	P	N	Random effects	I^2^(%)	*p*
Ethnicity							
Asia	454	900	22	400	16.90, 8.50–33.60	30.42%	0.1656
Africa	88	148	16	46	3.57, 0.10–126.16	92.66%	<0.0001
Source of sample							
Biopsies	227	338	16	89	15.32, 1.86–125.93	85.57%	<0.0001
Others	234	589	10	332	16.99, 8.91–32.40	0.00%	0.8971
Test method							
MSP	320	541	36	313	10.35, 2.71–39.53	84.43%	<0.0001
Others	222	507	2	133	25.31, 7.88–81.25	0.00%	0.4492

## Data Availability

Not applicable.

## References

[1] Chen Y.P., Chan A.T., Le Q.T., Blanchard P., Sun Y., Ma J. (2019). Nasopharyngeal Carcinoma. Lancet.

[2] Sung H., Ferlay J., Siegel R.L., Laversanne M., Soerjomataram I., Jemal A., Bray F. (2021). Global Cancer Statistics 2020: GLOBOCAN Estimates of Incidence and Mortality Worldwide for 36 Cancers in 185 Countries. CA Cancer J. Clin..

[3] Tan W.-L., Tan E.-H., Lim D.W.-T., Ng Q.-S., Tan D.S.-W., Jain A., Ang M.-K. (2016). Advances in systemic treatment for nasopharyngeal carcinoma. Chin. Clin. Oncol..

[4] Stan D.J., Niculet E., Lungu M., Onisor C., Rebegea L., Vesa D., Bezman L., Bujoreanu F.C., Sarbu M.I., Mihailov R. (2021). Nasopharyngeal carcinoma: A new synthesis of literature data (Review). Exp. Ther. Med..

[5] Moro J.D.S., Maroneze M.C., Ardenghi T., Barin L.M., Danesi C.C. (2018). Oral and oropharyngeal cancer: Epidemiology and survival analysis. Einstein.

[6] Tabuchi K., Nakayama M., Nishimura B., Hayashi K., Hara A. (2011). Early Detection of Nasopharyngeal Carcinoma. Int. J. Otolaryngol..

[7] Ahmed N., Abusalah M.A.H.A., Farzand A., Absar M., Yusof N.Y., Rabaan A.A., AlSaihati H., Alshengeti A., Alwarthan S., Alsuwailem H.S. (2022). Updates on Epstein–Barr Virus (EBV)-Associated Nasopharyngeal Carcinoma: Emphasis on the Latent Gene Products of EBV. Medicina.

[8] Xu Y., Chen L., Chen Y., Ye W., Huang X., Ke M., Ye G., Lin L., Dong K., Lin Z. (2022). Prediction of Potential Biomarkers in Early-Stage Nasopharyngeal Carcinoma Based on Platelet RNA Sequencing. Mol. Biotechnol..

[9] Futscher B.W. (2012). Epigenetic Changes During Cell Transformation. Adv. Exp. Med. Biol..

[10] Wu K., Xu X.-N., Chen Y., Pu X.-L., Wang B.-Y., Tang X.-D. (2015). RASSF1A Gene Methylation is Associated with Nasopharyngeal Carcinoma Risk in Chinese. Asian Pac. J. Cancer Prev..

[11] Truong K.P., Lao D.T., Le H.A.T. (2020). CDKN2A methylation—An Epigenetic Biomarker for Cervical Cancer Risk: A Meta-Analysis. Pharmacophore.

[12] Lao T.D., Truong P.K., Thieu H.H., Le T.A.H. (2020). The Prognosis Value of CDH-1 Methylation–The Epigenetic Biomarker in Nasopha-ryngeal Carcinoma: Systematic Review and Meta-Analysis. Asian J. Pharma. Res. Health Care.

[13] Lin Y., Hupp T.R., Stevens C. (2009). Death-associated protein kinase (DAPK) and signal transduction: Additional roles beyond cell death. FEBS J..

[14] Bialik S., Kimchi A. (2006). The Death-Associated Protein Kinases: Structure, Function, and Beyond. Annu. Rev. Biochem..

[15] Fendri A., Masmoudi A., Khabir A., Sellami-Boudawara T., Daoud J., Frikha M., Ghorbel A., Gargouri A., Mokdad-Gargouri R. (2009). Inactivation of RASSF1A, RARbeta2 and DAP-kinase by promoter methylation cor-relates with lymph node metastasis in nasopharyngeal carcinoma. Cancer Biol. Ther..

[16] Nawaz I., Moumad K., Martorelli D., Ennaji M.M., Zhou X., Zhang Z., Dolcetti R., Khyatti M., Ernberg I., Hu L.-F. (2015). Detection of nasopharyngeal carcinoma in Morocco (North Africa) using a multiplex methylation-specific PCR biomarker assay. Clin. Epigenetics.

[17] Zhang J., Shen Z., Liu H., Liu S., Shu W. (2018). Diagnostic potential of methylated *DAPK* in brushing samples of nasopharyngeal carcinoma. Cancer Manag. Res..

[18] Lao T.D., Nguyen T.N., Le T.A.H. (2021). Promoter Hypermethylation of Tumor Suppressor Genes Located on Short Arm of the Chromosome 3 as Potential Biomarker for the Diagnosis of Nasopharyngeal Carcinoma. Diagnostics.

[19] Reis R.S.D., Santos J.A.D., Abreu P.M.D., Dettogni R.S., Santos E.D.V.W.D., Stur E., Agostini L.P., Anders Q.S., Alves L.N.R., Valle I.B.D. (2020). Hypermethylation status of DAPK, MGMT and RUNX3 in HPV negative oral and oropharyngeal squamous cell carcinoma. Genet. Mol. Biol..

[20] Park G.-B., Jeong J.-Y., Kim D. (2019). Gliotoxin Enhances Autophagic Cell Death via the DAPK1-TAp63 Signaling Pathway in Paclitaxel-Resistant Ovarian Cancer Cells. Mar. Drugs.

[21] Tur M.K., Daramola A.K., Gattenlöhner S., Herling M., Chetty S., Barth S. (2017). Restoration of DAP Kinase Tumor Suppressor Function: A Therapeutic Strategy to Selectively Induce Apoptosis in Cancer Cells Using Immunokinase Fusion Proteins. Biomedicines.

[22] Higgins J.P.T., Thompson S.G. (2002). Quantifying heterogeneity in a meta-analysis. Stat. Med..

[23] Dersimonian R. (1996). Meta-analysis in the design and monitoring of clinical trials. Stat. Med..

[24] Higgins J.P., Thompson S.G., Deeks J.J., Altman D.G. (2003). Measuring inconsistency in meta-analyses. BMJ.

[25] Begg C.B., Mazumdar M. (1994). Operating characteristics of a rank correlation test for publication bias. Biometrics.

[26] Egger M., Smith G.D., Schneider M., Minder C. (1997). Bias in meta-analysis detected by a simple, graphical test. BMJ.

[27] Kwong J., Lo K.W., To K.F., Teo P.M., Johnson P.J., Huang D.P. (2022). Promoter hypermethylation of multiple genes in nasopharyngeal carcinoma. Clin. Cancer Res..

[28] Wong T.S., Chang H.W., Tang K.C., Wei W.I., Kwong D.L., Sham J.S., Yuen A.P., Kwong Y.L. (2002). High frequency of promoter hypermethylation of the death-associated protein-kinase gene in nasopharyngeal carcinoma and its detection in the peripheral blood of patients. Clin. Cancer Res..

[29] Tong J.H., Tsang R.K., Lo K.W., Woo J.K., Kwong J., Chan M.W., Chang A.R., van Hasselt C.A., Huang D.P., To K.F. (2002). Quantitative Epstein-Barr virus DNA analysis and detection of gene promoter hypermethylation in nasopharyngeal (NP) brushing samples from patients with NP carcinoma. Clin. Cancer Res..

[30] Chang H.W., Chan A., Kwong D.L., Wei W.I., Sham J.S., Yuen A.P. (2003). Evaluation of hypermethylated tumor suppressor genes as tumor markers in mouth and throat rinsing fluid, nasopharyngeal swab and peripheral blood of nasopharygeal carcinoma patient. Int. J. Cancer.

[31] Wong T.S., Kwong D.L., Sham J.S., Wei W.I., Kwong Y.L., Yuen A.P. (2004). Quantitative plasma hypermethylated DNA markers of undifferentiated nasopharyngeal carcinoma. Clin. Cancer Res..

[32] Kong W.J., Zhang S., Guo C.K., Wang Y.J., Chen X., Zhang S.L., Zhang D., Liu Z., Kong W. (2006). Effect of methylation-associated silencing of the death-associated protein kinase gene on nasopharyngeal carcinoma. Anticancer Drugs.

[33] Hutajulu S.H., Indrasari S.R., Indrawati L.P., Harijadi A., Duin S., Haryana S.M., Steenbergen R.D., Greijer A.E., Middeldorp J.M. (2011). Epigenetic markers for early detection of nasopharyngeal carcinoma in a high risk population. Mol. Cancer.

[34] Challouf S., Ziadi S., Zaghdoudi R., Ksiaa F., Ben Gacem R., Trimeche M. (2012). Patterns of aberrant DNA hypermethylation in nasopharyngeal carcinoma in Tunisian patients. Clin. Chim. Acta.

[35] Zhang Z., Sun D., Hutajulu S.H., Nawaz I., Nguyen Van D., Huang G., Haryana S.M., Middeldorp J.M., Ernberg I., Hu L.F. (2012). Development of a non-invasive method, multiplex methylation specific PCR (MMSP), for early diagnosis of nasopharyngeal carcinoma. PLoS ONE.

[36] Tian F., Yip S.P., Kwong D.L., Lin Z., Yang Z., Wu V.W. (2013). Promoter hypermethylation of tumor suppressor genes in serum as potential biomarker for the diagnosis of nasopharyngeal carcinoma. Cancer Epidemiol..

[37] Yang X., Dai W., Kwong D.L., Szeto C.Y., Wong E.H., Ng W.T., Lee A.W., Ngan R.K., Yau C.C., Tung S.Y. (2015). Epigenetic markers for noninvasive early detection of nasopharyngeal carcinoma by methylation-sensitive high resolution melting. Int. J. Cancer.

[38] Wajed S.A., Laird P.W., DeMeester T.R. (2001). DNA Methylation: An Alternative Pathway to Cancer. Ann. Surg..

[39] Lakshminarasimhan R., Liang G. (2016). The Role of DNA Methylation in Cancer. Adv. Exp. Med. Biol..

[40] Chen Q.W., Zhu X.Y., Li Y.Y., Meng Z.Q. (2013). Epigenetic regulation and cancer (Review). Oncol. Rep..

[41] Huang Z., Bassil C.F., Murphy S.K. (2013). Methylation-specific PCR. Methods Mol. Biol..

[42] Agodi A., Barchitta M., Quattrocchi A., Maugeri A., Vinciguerra M. (2015). DAPK1 Promoter Methylation and Cervical Cancer Risk: A Systematic Review and a Meta-Analysis. PLoS ONE.

